# A Bibliometric Analysis of Scientific Production on Second-Generation Anti-Psychotic Drugs in Malaysia

**DOI:** 10.21315/mjms2018.25.3.5

**Published:** 2018-06-28

**Authors:** Francisco López-Muñoz, Francisco J Povedano-Montero, Kok-Yoon Chee, Winston W Shen, Poveda Fernández-Martín, Javier García-Pacios, Gabriel Rubio, Cecilio Álamo

**Affiliations:** 1 Faculty of Health Sciences, University Camilo José Cela, Madrid, Spain; 2 Neuropsychopharmacology Unit, Hospital 12 de Octubre Research Institute (i+12), Madrid, Spain; 3 Portucalense Institute of Neuropsychology and Cognitive and Behavioural Neurosciences (INPP), Portucalense University, Porto, Portugal; 4 Thematic Network for Cooperative Health Research (RETICS), Addictive Disorders Network, Health Institute Carlos III, MICINN and FEDER, Madrid, Spain; 5 Faculty of Biomedical Sciences and Health, European University of Madrid, Spain; 6 Department of Psychiatry & Mental Health, Tunku Abdul Rahman Institute of Neuroscience, Kuala Lumpur Hospital, Kuala Lumpur, Malaysia; 7 Departments of Psychiatry, Wan Fang Medical Center and School of Medicine, Taipei Medical University, Taipei, Taiwan; 8 Laboratory of Cognitive and Computational Neuroscience, Center for Biomedical Technology, Technical University of Madrid and Complutense University of Madrid, Spain; 9 Department of Psychiatry, “Doce de Octubre” University Hospital, Complutense University, Madrid, Spain; 10 Department of Biomedical Sciences (Pharmacology Area), Faculty of Medicine and Health Sciences, University of Alcalá, Alcalá de Henares, Madrid, Spain

**Keywords:** second-generation anti-psychotics, atypical anti-psychotics, bibliometrics, schizophrenia, bipolar disorder, Malaysia

## Abstract

**Objective:**

We carried out a bibliometric study on the scientific papers related to second-generation antipsychotic drugs (SGAs) in Malaysia.

**Methods:**

With the SCOPUS database, we selected those documents made in Malaysia whose title included descriptors related to SGAs. We applied bibliometric indicators of production and dispersion, as Price’s law and Bradford’s law, respectively. We also calculated the participation index of the different countries. The bibliometric data were also been correlated with some social and health data from Malaysia (total per capita expenditure on health and gross domestic expenditure on R&D).

**Results:**

We found 105 original documents published between 2004 and 2016. Our results fulfilled Price’s law, with scientific production on SGAs showing exponential growth (*r* = 0.401, vs. *r* = 0.260 after linear adjustment). The drugs most studied are olanzapine (9 documents), clozapine (7), and risperidone (7). Division into Bradford zones yields a nucleus occupied by the *Medical Journal of Malaysia, Singapore Medical Journal, Australian and New Zealand Journal of Psychiatry, and Pharmacogenomics*. Totally, 63 different journals were used, but only one in the top four journals had an impact factor being greater than 3.

**Conclusion:**

The publications on SGAs in Malaysia have undergone exponential growth, without evidence a saturation point.

## Introduction

Schizophrenia, with prevalence of 0.5%–1.0% in the general population (1), has been listed as one of 10 leading disorders carrying high disability in the adult population since 2001 (2). In 1952, chlorpromazine was first discovered for treating schizophrenia (3, 4). Chlorpromazine and its related first-generation anti-psychotic (FGA or typical anti-psychotic) drugs have efficacy of positive symptoms of schizophrenia, but produces marked distressing extrapyramidal side effects (EPS). Consequently, clozapine (5) and related second-generation anti-psychotic (SGA, or atypical anti-psychotic) were introduced, showing efficacy for both positive and negative symptoms. Thus, the whole generation of SGAs have been rapidly marketed with first introduction of risperidone in 1993 (6).

Table 1 lists clinical development of SGAs in the past 20 years. Besides the better efficacy for positive and negative symptoms, SGAs have also improved the quality of life (QoL) of psychotic patients, and contributed to weakening the stigma in drug treatment with drugs (7). Since 1993, with the clinical advent of the new SGA and, later on, with their licensing in treating bipolar disorder in 2003, SGA research have been advanced remarkably, leading to a considerable increased SGA papers in scientific literature. In this bibliometric study, we are here focusing on the production of papers in Malaysia.

Malaysia is an upper middle income country situated in south-east Asia. Its population is 30.3 million in 2015, with more than 70% are urban population. Mental health expenditures by the Ministry of Health in 2011 are 0.39% of the total health budget (8). In 2013, Malaysia has 242 psychiatrists (24% of them are in private sector), with ratio of 1 psychiatrist for 123,400 population (9). Malaysia has 4 public mental hospitals, and 48 psychiatric units in general hospitals (10). National Mental Health Registry (NMHR) was set up in 2003 by the Ministry of Health Malaysia to collect information about people with mental illnesses in Malaysia (11). This information is then used to estimate the incidence of selected mental illness and to evaluate its risk factors and treatment in the country, in helping plan mental health service. Based on the NMHR Schizophrenia Report 2003–2005, the incidence was five cases per 100,000 population/year (11), in which the incidence was thought to be severely under-represented (12) if compared to 7.2–20.01 per 100,000,000 in the UK (11).

The bibliometric indicators are used to study research activity in a specific country or in a particular field, and the whole idea is based on the assumption that scientific publication is the essential result of such research activity (13). Bibliometric studies are useful tools to evaluate the social and scientific relevance of a given discipline or field (14). With a bibliometric approach, our group has studied the evolution of scientific literature in psychiatry by specific research groups, on different psychiatric disorders, on aspects related to the discipline, and on specific therapeutic tools in the field of psychopharmacology (15–19). Recently, we have also analysed SGA scientific production in different Asian countries (20–25), Australia (26), Spain (27), Asia-Pacific region (28), and the world (29). In this study, we intended to extend the SGA bibliometric information in studying publication papers on SGA in Malaysia.

## Methods

### Data Collection

This bibliometric study was to use the database of SCOPUS, which is the largest abstract and citation database of peer-reviewed literature—scientific journals, books and conference proceedings. SCOPUS covers about 22,000 titles from over 5,000 publishers, of which 20,000 are peer-reviewed journals in the scientific, technical, medical, and social sciences (including arts and humanities).

Using remote downloading techniques, we chose papers containing in the AD (author address) section the descriptor *Malaysia*, and in the TI (title) section, the descriptors atypic* anti-psychotic*, second-generation anti-psychotic*, clozapine, risperidone, olanzapine, ziprasidone, quetiapine, sertindole, aripiprazole, paliperidone, amisulpride, zotepine, asenapine, iloperidone, lurasidone, *perospirone* and *blonanserin*, always limiting the year of publication to the period 2004–2016. The rest of the descriptors, referring to pharmacological aspects, were not restricted to any field of the database. We included the documents published since 2004 because it is precisely from that date, when there is continuity in the publication of scientific articles on the subject treated in Malaysia. In this study, we considered all the original articles, brief articles, reviews, editorials, letters to the editor, etc., and all duplicated documents were eliminated. The protocol on this topic of study was approved by Ethics Committee for Human Subjects at Camilo José Cela University in Madrid, Spain, as a part of a project supported by the grant UCJC 2012-01.

### Bibliometric Indicators

A bibliometric indicator of production is Price’s law (30). This law, is widely used in analysing the productivity of a specific discipline or a particular country, reflects a fundamental aspect of scientific production, which is its exponential growth. To assess whether the growth of scientific production in SGA follows Price’s law of exponential growth, we made a linear adjustment of the data obtained, according to the equation *y =* 0.719*x–*1438.7, and another adjustment to an exponential curve, according to the equation *y =* 2*E–*11*e*^0.135^*^x^*.

Other quantities related to growth are doubling time and annual growth rate. The first is the amount of time required for the subject matter to double its production; the annual growth rate represents how the magnitude has grown over the previous year, expressed as a percentage. The equation to calculate the doubling time (D) is expressed by the following equation:

$$D=\frac{Ln2}{b}$$

Where *b* represents the constant that relates the rate of growth to the size of the science already acquired. To calculate the annual growth rate, we used the following equation:

$$R=100\;(e^{b}-1)$$

Lotka expressed the frequency distribution of scientific productivity by the number of published articles, also known as the “inverse square law of scientific production” (31). It analysed the publication volume of authors and found that the number of authors who publish fewer papers is greater than those who publish many (32). In mathematical terms, the original law is expressed by the formula:

$$A(n)=\frac{A(1)}{n^{2}}$$

According to this index, the authors are categorised into three levels of productivity: small producers (those who publish one article); medium-sized producers, (those who publish 2–9 articles); and large-scale producers (those who publish 10+ articles). To estimate the importance of the authors, we have used the *index h* (33). This indicator is to estimate the number of relevant papers published by an author, increasing the requirement to increases its value. This indicator is a simplification of use and is the only that combines production and impact; in addition, this indicator eliminate biases caused by the tails of distribution of appointments.

We also used Bradford’s law as a bibliometric indicator for the dispersion of scientific information (34). This law is to show the distribution of the scientific literature in a particular discipline, and Bradford proposed a model of concentric zones of productivity (Bradford zones) with decreasing density of information. Thus, each zone would contain a similar number of documents, but the number of journals in which these are published would increase on passing from one zone to another. This model is used to identify the most widely used journals with greatest weight in a given field of scientific production.

We used impact factor (IF) as an indicator of the publication’s repercussion (35). This indicator was developed at the Institute for Scientific Information (Philadelphia, Pennsylvania, USA), in its annual publication of the Journal Citation Reports (JCR) section of the Science Citation Index (SCI). The IF of a journal is calculated on the basis of the number of times this journals is cited in the source journals of the SCI during the two previous years and the total number of articles published by the journal in question in those two years. The JCR lists scientific journals by specific areas, ascribing to each of them their corresponding IF and establishing a ranking of “prestige.” We used the IF data of 2015 published in the JCR.

We also included national participation index (PI) of Malaysia as another indicator included in this study by calculationg the ratio of the number of papers generated by Malaysia and the total number of documents on this topic. This PI has also been compared with global PI in biomedical and health sciences (as well as for Psychiatry and Mental Health area in particular). Likewise, the PI has been correlated with some health data, such as total per capita expenditure on health and gross domestic expenditure on R&D. The PI has also been correlated with the corresponding PI for the world’s 10 most productive countries during the period 1996–2015. The health data were obtained from The World Bank (10) and World Health Organisation Department of Health Statistics and Informatics (36).

## Results

After studying analysed database during the period 2004–2016, we identified 105 original papers (articles, reviews, editorials, letters to the editor, etc.) dealing with different aspects related to SGAs in Malaysia. Of them, nine corresponded to olanzapine, seven to clozapine, seven to risperidone, six to ziprasidone, six to quetiapine, six to paliperidone, five to aripiprazole, and one to asenapine. We did not find any papers which were relative to sertindole, amisulpride, zotepine, iloperidone, lurasidone, perospirone, and blonaserin.

**Figure 1:**
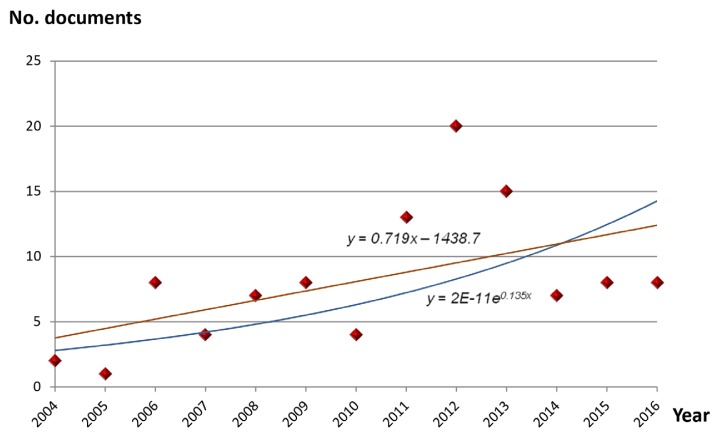
Growth of scientific production on SGAs in Malaysia. A linear adjustment of the data was carried out, and a fitting to an exponential curve, in order to check whether production follows Price’s law of exponential growth Linear adjustment: *y* = 0.719*x*–1438.7 (*r* = 0.027) Exponential adjustment: *y* = 2E–11e^0.135^*^x^* (*r* = 0.421)

As seen in Figure 1, over the last 13 years, there has been an increased number of papers related to SGAs in Malaysia at a worldwide basis. We got the mathematical adjustment to an exponential curve (Figure 1), with a correlation coefficient *r* = 0.421, indicating 57.9% of variance unexplained by this fitting. In contrast, the linear adjustment of the measured values provides an *r* = 0.279, and therefore a percentage of unexplained variance of 72.1%. With these data we conclude that the analysed database is more in keeping with an exponential fitting than a linear one, and postulated that Price’s law was fulfilled. But to bear in mind that the high degree of variability that there is, as the correlation coefficient is well below 1.

**Figure 2:**
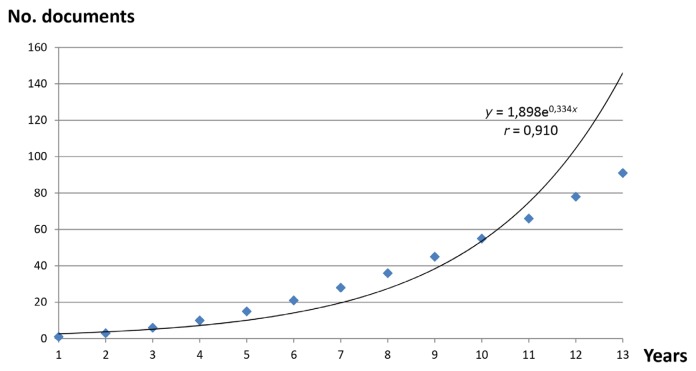
Temporal evolution of number of documents on second-generation anti-psychotic drugs (13 years)

To calculate doubling time, the scatter plot in Figure 2 shows the temporal production of publications along the trend line, which was fitted to the equation *y =* 1.898*e*^0.3314^*^x^*, with a correlation coefficient of 0.910. This production corresponds to 13 years and a doubling time of 2.06 years.

**Figure 3:**
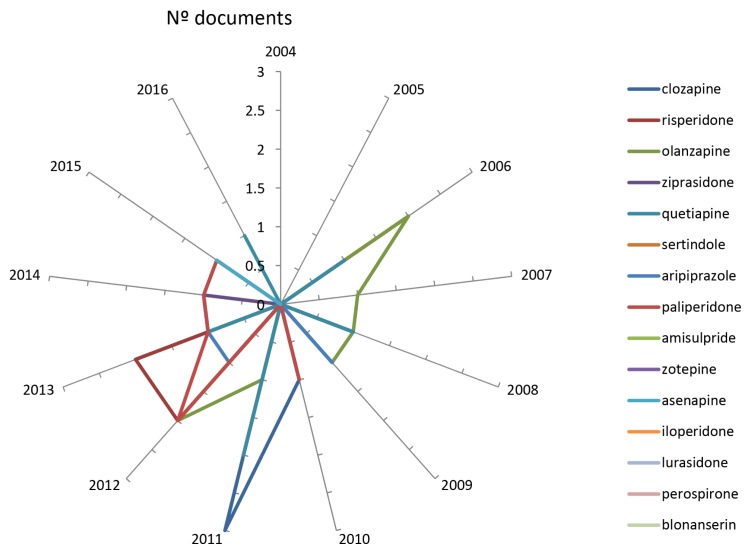
Number of papers on second-generation anti-psychotic drugs (2004–2016)

**Figure 4:**
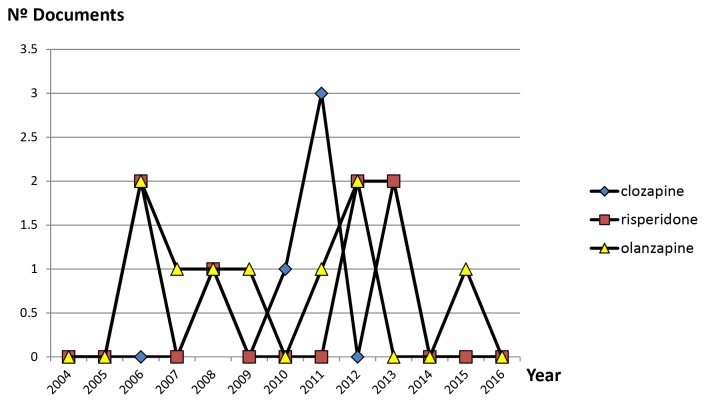
Evolution of documents on three more relevant second-generation anti-psychotic drugs

**Figure 5:**
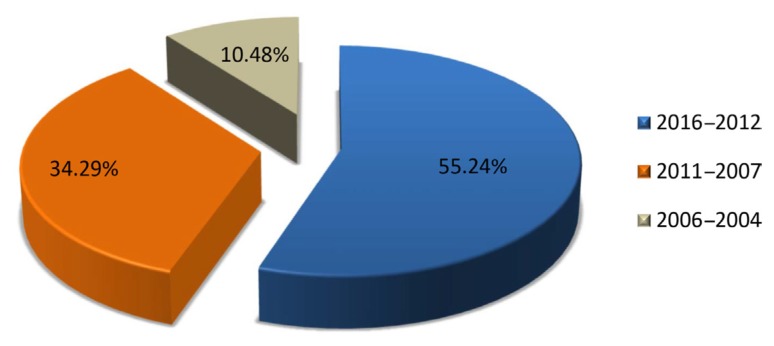
Evolution of the number of documents every three-years period

**Figure 6:**
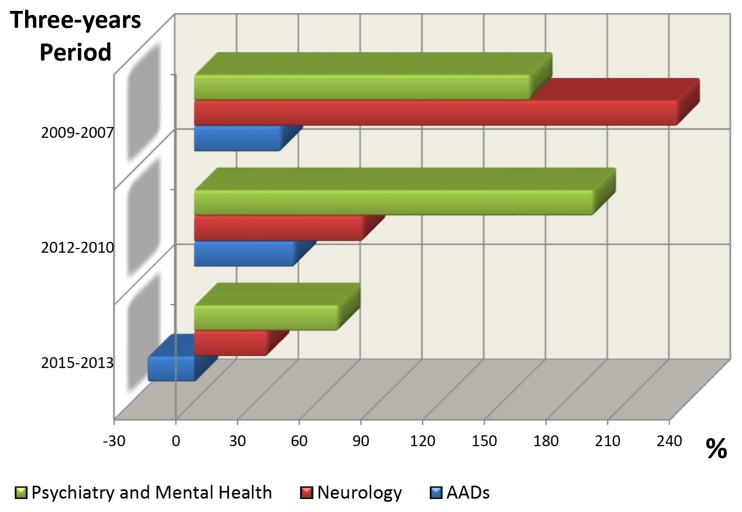
Cumulative growth by three-year periods of scientific production on total productivity in Psychiatry (and Mental Health) and Neurology area, and SGAs in Malaysia. Data from each three-year period refer to evolution over the previous period. The period of reference is 2004–2006. Data are expressed in percentages AADs: atypical anti-psychotic drugs, or SGA: second-generation antipsychotics

The clinical introduction of the new SGAs in different countries of the world, together with their authorisation for the treatment of bipolar disorder, is to have contributed substantially to the increase in scientific production in the field of SGAs in Malaysia (Figure 3). Figure 4 illustrates the evolution that has occurred in the last 13 years of the literature of the three most important SGAs. With effect from 2008, the growth was manifested, due mainly to two drugs, aripiprazole and olanzapine, respectively. Figure 5 shows the matter even better that that cumulative growth in total scientific production related to SGAs in Malaysia in each three-year period over the previous one even gradually increased. In Figure 6 is related to growth, for periods of three years, of the Psychiatry and Mental Health, the Neurology and SGAs.

After applying the Lotka law, the distribution of the authors was heavily concentrated in small producers, with a high index of transience (occasional authors) of 86.13 (Table 2). The total number of authors for 105 papers was 483, representing an index of co-authorship of 4.6. Among the most productive authors, there was great variability in the *index h*, ranging between 5 and 31 (Table 3).

In the scientific journals where the papers on SGAs have been published, we applied the Bradford’s model. The mean number of articles per Bradford zone was 35, even we discarded the last zone, whose accuracy was obviously lower, the mean would be 21. Table 4 shows the division into Bradford’s areas of the material under study. We found that 63 different journals were published in the analysed database, and the core consisted of 4 journals, *Medical Journal of Malaysia, Singapore Medical Journal, Australian and New Zealand Journal of Psychiatry* and *Pharmacogenomics*, of which only one had impact factor greater than 3 (Table 5). The preferential selections of journals may have the relationship of Malaysian colonial history in the past.

**Figure 7:**
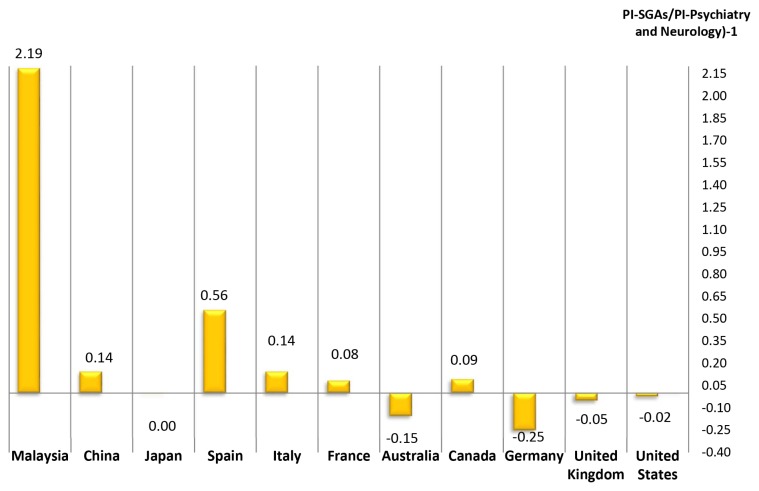
Relationship between production of scientific literature on SGAs and total production in the field of Psychiatry and Neurology in the world’s 10 most productive countries and Malaysia for the period 1996–2015 PI: participation index; SGAs: second-generation anti-psychotics

**Figure 8:**
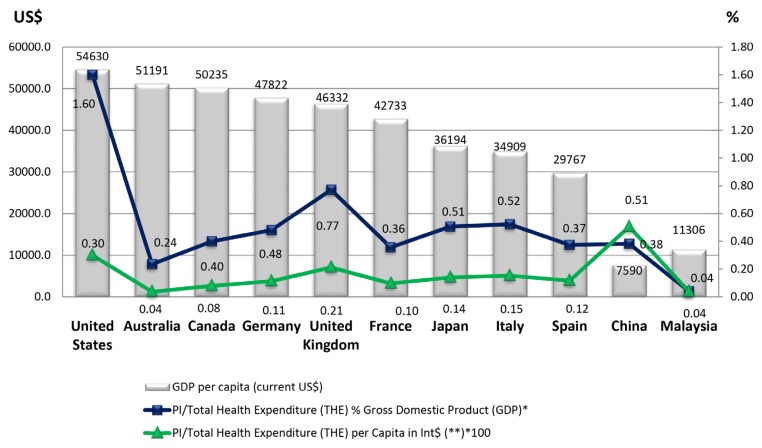
GDP per capita and relationship between production of scientific literature on SGAs and the total expenditure on health on gross domestic product and per capita in Int$, in the world’s 10 most productive countries in psychiatry and neurology and Malaysia *World Bank data (2014); **World Health Organization data (2015); GDP: gross domestic product; PI: participation index

The general contribution of Malaysia science, within this topic area, represents a global participation index of 0.22 with respect to world production in period analysed. Among the countries generating research on SGAs, the most significant, as Table 6 shows, is the United States, whose PI is 29.14, followed by the United Kingdom (PI = 7.94), Germany (PI = 5.93) and Japan (PI = 5.25). But if we consider the productivity of these countries in this area in relation to their overall production in the field of Psychiatry and Neurology, only 2 (Malaysia and Spain) of the 10 largest producers in the period 1996–2015, devote a higher percentage of attention to the study of SGAs (Figure 7). In the analysis of the correlation between PI and the per capita health expenditure of each of the countries with the highest scientific production in health sciences, the distribution obtained is quite similar (Figure 8).

Table 7 shows the most productive institutions in relation to the material under study. We found that more than 50% of total production was generated in 3 institutions (University of Malaya, Universiti Kebangsaan Malaysia [or National University of Malaysia], and Kuala Lumpur Hospital*)*. To note, we defined the corresponding institution solely based on the information given in the AD field in the SCOPUS web database.

## Discussion

Bibliometric studies have interesting tools for assessing the social and scientific importance of a given discipline over a specific time period. The term “bibliometrics” was introduced in 1969 by Alan Pritchard (37), to define the application of mathematical and statistical methods to the process of dissemination of written communication in the area of scientific disciplines, by means of quantitative analysis of the different aspects of this type of communication. Despite their methodological limitations, those analyses have given us an overview of the growth, size and distribution of the scientific literature related to a particular discipline, and the study of the evolution of not only the biomedical speciality, field of specialisation or issue in question, but also the scientific production of an institution, country, author or research group (13).

Previous bibliometric studies have been warned to have many limitations in doing the sociometric approach (38). Obviously, the international scientific production in a particular field such as SGAs in this study, is much more extensive (involving many journals or contributions made to scientific conferences and meetings are not indexed in the usual databases). But the recognised quality of the publications included in the databases used in this study and their coverage means that the papers chosen constitute a more than representative sample of the international research on the area in question.

Taking into account those assumptions, the design of the present analysis still allows us to make a global assessment of the growth of scientific literature related to SGAs in Malaysia. In this regard, Figure 1 shows, the number of scientific publications has been through an exponential growth over the last 13 years, and especially after 2012, up to the end of the period studied, without evidence of approaching saturation as postulated by Price in his theory on expansion of scientific literature (30). This observation should be seen as positive, that the time of duplication of the SGAs about scientific literature is situated in only 2.06 years, demonstrating the great dynamism for scientific activity in Malaysia.

The pharmacoepidemiological study of the Research on Asian Psychotropic (REAP) project (39, 40) in the region (China, Hong Kong, Japan, Korea, Taiwan, and Singapore) collected the data which have been highly correlated to the bibliometric data in this bibliometric study. The data of the REAP prescribing patterns of 2001, 2004, and 2008 have remarkable increased SGA uses of 45.5, 64.7% and 76.6%, respectively. Data from NMHR has also shown substancial increase in SGA uses among first-episode schizophrenia patients in Malaysia after a one-year follow-up (41). Based on unpublished data from the NMHR, prescription of oral FGAs has reduced from nearly 80% from all first-episode schizophrenia patients in 2003 to 20% in 2012, while the prescription of SGAs has been increased from 22% in 2003 to 78% in 2012. The number of SGA prescriptions has surpassed FGA prescriptions since 2009. Among the SGAs, prescription of risperidone has increased almost exponentially 18% in 2003–60% in 2012. This is likely due to the availability of generic form of risperidone especially in government reimbursement “blue book” (formularly) in Malaysia since 2009. Prescription formulary of other SGAs such as quetiapine, aripiprazole, amisulpride, and paliperidone were increased only slightly compared to risperidone. There was hardly any change in the prescription trend in clozapine in first episode schizophrenia from 2003 to 2012, remaining at around 2%–3%.

The development of the SGA scientific literature coincides with its approval for marketing by the US Food and Drug Administration (FDA) and other international regulatory agencies in treating bipolar disorder. Since 2004, other SGAs (such as risperidone, quetiapine, ziprasidone, aripiprazole, and asenapine, etc.) have been also approved for treating manic episodes, and olanzapine and aripiprazole for relapse prevention in patients with bipolar disorder. Quetiapine is indicated as monotherapy for the acute treatment of depressive episodes associated with bipolar disorder, and olanzapine-fluoxetine combination for treating treatment-resistant major depressive disorder. Aripiprazole was also approved in 2007 by the US FDA for treating treatment-resistant major depression as an add-on to an existing antidepressant. Finally, SGAs are also commonly used (and studied) in numerous off-label indications, such as toxicological psychosis, agitation symptoms, tics, substance abuse disorders, etc. (42–44).

We used the indicators of impact and excellence of the publications to analyse quality of published papers. As shown in Table 5, we found that four of the 10 journals were more productive with IF being greater than 2 (*Australian and New Zealand Journal of Psychiatry, Drug Safety, Pharmacogenomics* and *Human Psychopharmacology).* To note, *Medical Journal of Malaysia* published 5.17% of published Malaysian SGA articles, but this journal does not have any IF.

During the last 10 years, there had been an increase in scientific production in the area of Psychiatry and Neurology in Malaysia (Figure 6), with this percentage even higher in SGAs, at the height of other biomedical disciplines (Table 8). As we have shown in recent studies (20–26, 28), research scientific on SGAs is one of the fields experiencing faster growth with in psychiatry. Similarly, other authors using bibliometric tools, have reported that the research activity in the field of schizophrenia is superior to that of other psychiatric fields (45). These authors also suggest that the attraction of research on schizophrenia may have been positively affected by the clinical perception of the greater seriousness of the illness compared to other psychiatric pathologies. Theander and Wetterberg in 2010 (46) also report that the number of references on schizophrenia in Medline has followed the general increase of medical publications, accounting for 0.42% increase compared to that of the total medical literature in the period studied.

The two major English-speaking countries (United States and United Kingdom), heads the ranking of producer countries and between them have generated over a third of total scientific production in this field (37.08%). Those two countries are home of the pharmaceutical companies responsible for the development of SGAs (olanzapine–Eli Lilly, USA; risperidone and paliperidone–Janssen Pharmaceutica, USA; quetiapine–AstraZeneca, UK; ziprasidone–Pfizer, USA; and aripiprazole–Bristol-Myers Squibb/Otsuka Pharmaceutical Co., USA/Japan) may help explain their high PI.

Table 6 shows the data from the 11 most productive countries (and Malaysia) in the Psychiatry and Mental Health and Neurology discipline and compares them with productivity in the specific field of SGAs. It is worthy of note how some places, such as Germany, Italy, and Japan sit near the top of the ranking for SGAs production (Table 6), reflecting the special interest of those countries in studying those drugs. Other countries, such as the United States and United Kingdom maintain rates of productivity in SGAs research that are in proportion with their global index for psychiatry and neurology. Finally, there’s that highlight countries, such as Malaysia (especially in this country) and Spain, in the proportion of SGAs on these areas is greater (Figure 7).

The correlation of scientific production in SGAs with the per capita health expenditure of each country, shown in Figure 8, offers us a parallel view of this phenomenon; in general, there is confirmation of the notion that the higher the spending on health, the greater the research production. In this regard, it should be made clear that a country’s scientific production in a given field tends to reflect a science research and development policy begun some years prior to the period analysed, and is not the fruit of particular economic circumstances (17, 18). The establishment of NMHR for schizophrenia in 2003 by Ministry of Health Malaysia (12) and participation in REAP study since 2008 have contributed significantly in research production in the field of schizophrenia (39, 40). Malaysia is among the few countries that has registry for schizophrenia (11). The NMHR database contained clinical information of the person with schizophrenia, medical data including past and present medical illnesses, prescription data, pathway to care, etc. (12). The database has catalysed many research activities and publications.

It is striking to observe the low ratios of countries such as Australia, Canada or France, despite being recently the world’s first 7 to meet the gross domestic product (GDP) per capita*.* Malaysia, as a middle income country, has health expenditure relatively low (ranking 161; 4.17% of GDP) (8, 47). Also there is no insurance coverage for psychiatric illness (11). Mental health expenditures by the Ministry of Health in 2011 are 0.39% of the total health budget (8). The correlation analysis between scientific production in SGAs and the gross domestic in health expenditure located to Canada, Australia and Malaysia at the last three positions.

Despite the limitations inherent to the use of a single database for the development of this work, we have used SCOPUS for being one of the most comprehensive and easy database to use in biomedical field compared to any other tool for literature research. SCOPUS is considered as the world’s largest database for abstract and citation information (48) that regularly is used in bibliometric studies. In summary, we through this biblimetric study, can offer a representative picture of evolution of international research on SGAs in Malaysia, observing the parameters of quality and dissemination most commonly used at an international level. Research in this field will possibly continue to grow in the coming years, bearing in mind that the ideal antipsychotic drug has not yet been found (7) and that pathoetiology of schizophrenia is mainly still unknown. Besides, SGAs have, and will continue to have, an ever-expanding range of clinical indications, both within the psychiatric ambit and outside it, to judge from the promising results obtained for the different pathologies with which they have been studied. Thus, SGA scientific production in Malaysia will certainly continue to be abundant, as is happening with the prescription patterns of SGAs in this region.

## Figures and Tables

**Table 1:** Clinical development of SGAs

SGAs	Company	Launch	Country
Clozapine	Wander Laboratories	1972§	Suiza
Zotepine	Fujisawa	1982†	Japan
Amisulpride	Synthelabo	1986	Portugal
Risperidone	Johnson & Johnson	1993	UK/Canada
Sertindole	Abbott Laboratories	1996‡	UK
Olanzapine	Eli Lilly	1996	USA/UK
Quetiapine	AstraZeneca	1997	USA/UK
Ziprasidone	Pfizer	2001	USA
Perospirone	Dainippon Sumitomo Pharma	2001	Japan
Aripiprazole	Otsuka/Bristol-Myers Squibb	2002	USA
Paliperidone	Janssen Pharmaceutica	2007	USA
Blonanserin	Dainippon Sumitomo Pharma	2008	Japan
Asenapine	Schering-Plough	2009	USA
Iloperidone	Novartis AG	2009	USA
Lurasidone	Dainippon Sumitomo Pharma	2011	USA

§ Reintroduced in 1990 in the USA and the UK after being withdrawn from the market in 1975

† Commercialised by Astellas in Germany in 1990

‡ Marketing authorisation was suspended by the European Medicines Agency (EMA) in 1998 and the drug was withdrawn from the market. In 2002, based on new data, the EMA suggested that sertindole could be reintroduced for restricted use, and with extensive ECG monitoring requirement

**Table 2:** Author dispersion according to productivity level

	PI ≥ 1 (10 or more articles)	0 < PI < 1 (2–9 articles)	PI = 0 (1 article)	Total
Number of authors (%)	0 (0.00)	67 (13.87)	416 (86.13)	483 (100.00)

PI: participation index

**Table 3:** Analysis of the authors with more productive documents

Author	No. of documents	(%)	h-index	Affiliation
Chee KY	8	(7.62)	8	Kuala Lumpur Hospital, Department of Psychiatry and Mental Health, Malaysia
Chong MY	8	(7.62)	19	Chang Gung University, Department of Psychiatry, Kaohsiung, Taiwan
Chung EK	8	(7.62)	12	National Seoul Hospital, Department of Psychiatry, Seoul, South Korea
Fujii S	8	(7.62)	12	Fukushima Medical University, Japan
He YL	8	(7.62)	36	Shanghai Mental Health Center, Department of Psychiatriy, Shanghai, China
Kua EH	8	(7.62)	29	Yong Loo Lin School of Medicine, Department of Psychological Medicine, Singapore
Lee EHM	8	(7.62)	12	The University of Hong Kong, Pokfulam
Sartorius N	8	(7.62)	39	Association for the Improvement of Mental Health Programmes, Switzerland
Shinfuku N	8	(7.62)	20	Kobe University School of Medicine, Kobe, Japan
Si TM	8	(7.62)	17	Peking University, Key Laboratory of Mental Health, Beijing, China
Sim K.	8	(7.62)	23	National University of Singapore, Department of Pharmacology, Singapore
Tan CH	8	(7.62)	23	National University of Singapore, Department of Pharmacology, Singapore
Trivedi JK	8	(7.62)	13	Chhatrapati Shahuji Maharaj Medical University, Department of Psychiatry, India
Udomratn P	8	(7.62)	10	Prince of Songkla University, Department of Psychiatry, Thailand
Ungvari GS	8	7.62)	31	University of Notre Dame, Australia
Wang CY	8	(7.62)	20	Capital Medical University, Center of Schizophrenia, China
Yang SY	8	(7.62)	13	Taipei City Hospital, Taipei, Taiwan
Xiang YT	8	(7.62)	21	University of Macau, Faculty of Health Science, Macau
Chiu HFK	7	(6.67)	9	Chinese University of Hong Kong, Department of Psychiatry, Hong Kong
Yong MKH	6	(5.71)	5	Singapore Institute of Mental Health, Singapore
Zainal N	6	(5.71)	10	University of Malaya, Department of Psychological Medicine, Malaysia
Mohamed Z	5	(4.76)	15	University of Malaya, Department of Pharmacology, Malaysia

**Table 4:** Distribution of the journals in Bradford’s zones

	No. of journal	% of journals	No. of articles	% of articles	Bradford multiplier
Core	4	5.00%	16	15.24%	
Zone 1	13	16.25%	26	24.76%	3.3
Zone 2	63	78.75%	63	60.00%	4.8
Total	80	100.00%	105	100.00%	4.1

Total number of journals = 80

Average number of articles = 35

Average number of articles, excluding the last Bradford’s zone = 21

**Table 5:** The four journals with highest number of publications on second-generation anti-psychotics

Source	No. of documents	PI	Impact Factor*	Eigenfactor® Score	Article Influence® Score	Country of origin
*Medical Journal of Malaysia*	6	5.71				Malaysia
*Singapore Medical Journal*	4	3.81	0.573	0.00317	0.206	Singapore
*Australian and New Zealand Journal of Psychiatry*	3	2.86	3.536	0.00902	1.120	Australia
*Pharmacogenomics*	3	2.86	2.710	0.00803	0.818	United Kingdom

PI: participation index; IF: impact factor 2015.

* Journal Citation Report

**Table 6:** Distribution of documents on SGAs, Psychiatry and Neurology in the world’s 11 most productive and Malaysia for the period 1996–2015

Country^1^	Psychiatry and Mental Health^2^ %	Neurology^3^ %	SGAs^4^ %	SGAs/Psy-Neurol
United States	34.26	27.33	29.14	0.98
United Kingdom	10.78	7.01	7.94	0.95
Germany	7.16	8.34	5.93	0.75
Canada	4.85	4.34	4.94	1.09
Australia	4.26	2.56	2.68	0.85
France	3.95	4.32	4.53	1.08
Italy	3.59	5.53	5.53	1.14
Netherlands	3.15	2.70	2.40	0.84
Spain	2.32	2.80	4.09	1.56
Japan	2.07	7.06	5.25	1.00
China	1.17	3.21	2.84	1.14
Malaysia	0.10	0.09	0.22	3.19

Psy-Neurol (area of focus in Neurology and Psychiatry); SGAs (second-generation anti-psychotics)

1 The world’s 11 most productive countries in Psychiatry (and Mental Health) and Neurology for the period 1996–2015

2 Total documents 1996–2015 in Psychiatry and Mental Health: 544,264 (Scimago Journal & Country Rank)

3 Total documents in Neurology 1996–2015: 968,287 (Scimago Journal & Country Rank)

4 Total documents SGAs 1996–2015: 45,745

**Table 7:** Contribution of different institutions

Centre	n	PI
University of Malaya	34	32.38
Universiti Kebangsaan Malaysia	15	14.29
Kuala Lumpur Hospital	13	12.38
School of Medical Sciences, Universiti Sains Malaysia	10	9.52
Chinese University of Hong Kong	9	8.57
Association for the Improvement of Mental Health Programs	8	7.62
Hyogo Institute for Traumatic Stress HITS	8	7.62
Prince of Songkla University	8	7.62
Peking University	8	7.62
National University of Singapore	8	7.62
Seinan Gakuin University	8	7.62
Chhatrapati Shahuji Maharaj Medical University	8	7.62
Taipei City Hospital Taiwan	8	7.62
Beijing An Ding Hospital, Capital Medical University	8	7.62

*n*: number of documents of database

PI: participation index

**Table 8:** Contribution of Malaysia in different areas of biomedicine

Area	Rank*	PI
Medicine	43	0.23
Biochemistre, Genetics and Molecular Biology	41	0.25
Health Professions	40	0.18
Neuroscience	47	0.08
Pharmacology, Toxicology and Pharmaceutics	37	3.67
Psychiatry and Mental Health	47	0.10
Neurology	55	0.04
Neurology (Clinical)	61	0.03

* Data Scimago Journal and Country Rank (1996–2015)

PI: participation index
